# Isolated cryptococcal pleural effusion in a heart transplant recipient: A case report and literature review of pleural fluid adenosine deaminase levels

**DOI:** 10.1002/rcr2.1297

**Published:** 2024-02-16

**Authors:** Jaehee Lee, Bo Eun Park, Se Yong Jang, Chang Ho Kim

**Affiliations:** ^1^ Department of Internal Medicine Kyungpook National University, School of Medicine Daegu Republic of Korea

**Keywords:** adenosine deaminase, cryptococcal antigen, cryptococcal pleural effusion

## Abstract

Isolated cryptococcal pleural effusion is rare as the initial clinical presentation in opportunistic cryptococcal infection. We describe a 59‐year‐old male heart transplantation recipient who presented with a mononuclear‐leukocyte‐predominant exudative pleural effusion, with adenosine deaminase levels (ADA) of 37 IU/L and focal pleural nodularity on computed tomography. A thorough evaluation, including pleural fluid culture, cryptococcal antigen, and histological examination, led to the diagnosis of cryptococcal pleural effusion. Antifungal therapy with fluconazole of 400 mg/day showed clinical and radiological improvement. A literature review identified six cases of cryptococcal pleural effusion that reported pleural fluid ADA levels. All cases, including the present one, involved immunocompromised hosts and exhibited a mononuclear‐leukocyte‐predominant exudate. High pleural fluid ADA levels were observed in approximately half of these cases. The pleural fluid cryptococcal antigen test was an important diagnostic tool for early diagnosis. In an era where immunocompromised hosts are increasing, cryptococcal infection should be considered as a potential aetiology in immunosuppressed patients with an exudative pleural effusion of unknown cause, even if ADA levels are elevated.

## Graphical abstract

We report a case of isolated cryptococcal pleural effusion and review its characteristics with reference to pleural fluid adenosine deaminase (ADA) levels.

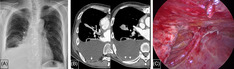

## INTRODUCTION

Isolated pleural effusion has rarely been reported as the initial clinical presentation in opportunistic cryptococcal infection.^1^, ^2^ In an era marked by a growing number of immunosuppressed patients, it is crucial to consider opportunistic infections as an alternative cause for early diagnosis and treatment in immunocompromised patients with pleural effusion of unknown origin. After the measurement of pleural fluid adenosine deaminase (ADA) levels became common in the 1990s, a few cases of cryptococcal pleural effusion reported their pleural fluid ADA levels, and elevated ADA levels have often been reported among these cases.^2^, ^3^, ^4^, ^5^, ^6^, ^7^ These characteristics of cryptococcal pleural effusion pose challenges in differentiating it from tuberculous pleural effusion (TPE), especially in regions with a high to intermediate tuberculosis prevalence. Some prior cases of cryptococcal pleural effusion have resulted in unnecessary anti‐tuberculosis therapy.^5^, ^6^ Pleural fluid ADA levels have not been previously reviewed in the context of cryptococcal pleural effusion. Thus, we report a case of isolated cryptococcal pleural effusion and review its characteristics with reference to pleural fluid ADA levels.

## CASE REPORT

A 59‐year‐old man, who had undergone heart transplantation 7 months earlier due to severe ischemic cardiomyopathy resulting from acute myocardial infarction, was admitted to the cardiology division due to right pleuritic chest pain lasting for 4 days. Immunosuppressive regimens, including tacrolimus, mycophenolic acid, and prednisolone have been administered since his cardiac transplantation. On vital signs, blood pressure was 120/80 mmHg, heart rate 70 beats/min, respiratory rate 22/min, and body temperature 37.3°C. Blood white blood cell (WBC) count was 8460/uL, serum C‐reactive protein 1.84 mg/dL, serum total protein 5.8 g/dL, serum lactate dehydrogenase (LDH) 286 U/L, and serum creatinine 1.29 mg/dL.

**FIGURE 1:**
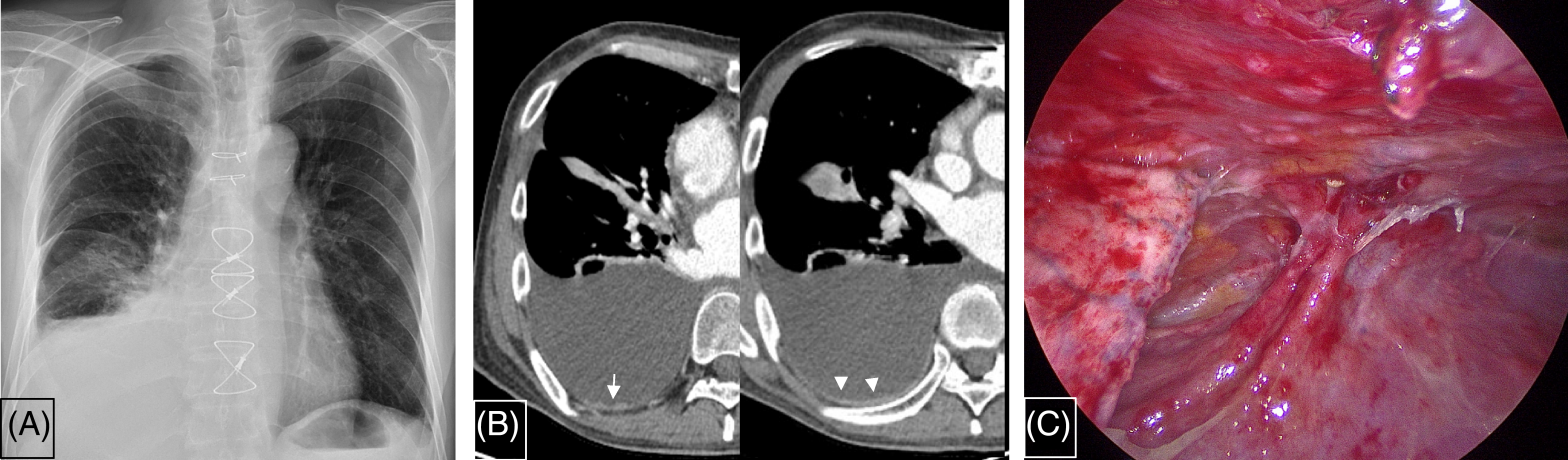
(A) Chest radiograph showed right‐sided pleural effusion. (B) Chest CT demonstrated right‐sided pleural effusion, diffuse pleural thickening (arrow head), and bumpy focal pleural thickening (arrow). (C) Thoracoscopic view of pleural cavity revealed multiple small nodules on the costal and diaphragmatic pleura.

Chest x‐ray revealed homogenous haziness in the right lower lung field (Figure 1A) and chest computed tomography (CT) showed a moderate amount of pleural effusion with diffuse pleural thickening, small‐sized, bumpy focal pleural nodularity, and subpleural passive atelectasis in the right lower lobe and middle lobe lateral segment (Figure 1B). Therapeutic pleural drainage was performed with empirical antibiotics. The analysis of pleural fluid showed a serous colour, WBC count of 741/uL with a mononuclear‐leukocyte (MN) predominance (62.4%), protein 3.0 g/dL, LDH 166 U/L, ADA 14.7 IU/L, and carcinoembryonic antigen 1.6 ng/mL (Table 1). Acid‐fast bacilli stain and polymerase chain reaction for *Mycobacterium tuberculosis* were negative. The cytology of the pleural fluid was negative. With symptomatic and radiological improvement, he was discharged 9 days later. One day after discharge, he developed a fever of 37.6°C and right pleuritic chest pain, leading to his readmission. Chest x‐ray revealed recurrent pleural effusion in the right hemithorax. It was noted that the initial fluid culture grew *Cryptococcus neoformans* on the 11th days after the first thoracentesis. The patient was referred to the pulmonary division. Repeated diagnostic thoracentesis now revealed an exudate with WBC count 501/uL, MN predominance (74.5%), protein 3.8 g/dL, LDH 132 U/L, ADA 22.7 IU/L (Table 1). The serum and pleural fluid cryptococcal antigen tests were negative, and the pleural fluid smear and culture were negative for all microorganisms.

**TABLE 1:** Review of pleural fluid analysis in previous cases of cryptococcal pleural effusion with pleural fluid adenosine deaminase levels.

Author(year ) (reference)	Age/sex	Predisposing condition	Disease presentation	Pleural fluid							Serum cryptococcal Ag	Treatment	Outcome
				TLC	Predominant cell	Protein (g/dL)	LDH (U/L)	ADA (IU/L)	Cryptococcal Ag	Cryptococcal culture			
Fukuchi (1998)^3^	52/F	RA/Steroid/ESRD‐HD	Localized	NR	MN	2.5	364	28	+	+	+	AMB/5FC/FLU	Recovered
Kinjo (2009)^4^	64/M	ESRD‐HD	Localized	2100	MN (Ly‐94%)	5.4	264	33	+	+	−	AMB/5FC/FLU	Recovered
Yoshino (2010)^5^	51/M	HIV‐positive	Localized	2600	NR	NR	NR	86	NR	+	+	AMB/FLU	Recovered
Chen (2015)^2^	63/M	Renal transpl.	Disseminated	920	MN (Ly‐70%)	4.1	121	24/25	NR	−	+	AMB/5FC/VOR/FLU	Recovered
Wee (2018)^6^	38/M	AML	Localized	4150	MN (Ly‐81%)	6.1	241	53/51	NR	+	−	AMB/5FC/FLU	Recovered
Kushima (2018)^7^	80/M	RA/Steroid	Localized	400	MN (Ly‐100%)	NR	NR	101	NR	+	+	FLU	Recovered
Present case (2023)	59/M	Heart transpl.	Localized	741/501/304	MN (62/75/97%)	3.0/3.8/4.1	166/132/148	15/23/37	−/+	+/−/−	−/+	FLU	Recovered

*Note*: / indicates the separate performance of two or three times.

Abbreviations: ADA, adenosine deaminase; Ag, antigen; AMB, amphotericin‐B; AML, acute myeloid leukaemia; ESRD‐HD, end‐stage renal disease‐haemodialysis; FLU, fluconazole; HIV, human immunodeficiency virus; LDH, lactate dehydrogenase; Ly, lymphocyte; MN, mononuclear leukocyte; NR, not reported; RA, rheumatoid arthritis; TLC, total leukocyte count; Transpl, transplantation; VOR, voriconazole; 5FC, 5‐flucytosine.

**FIGURE 2:**
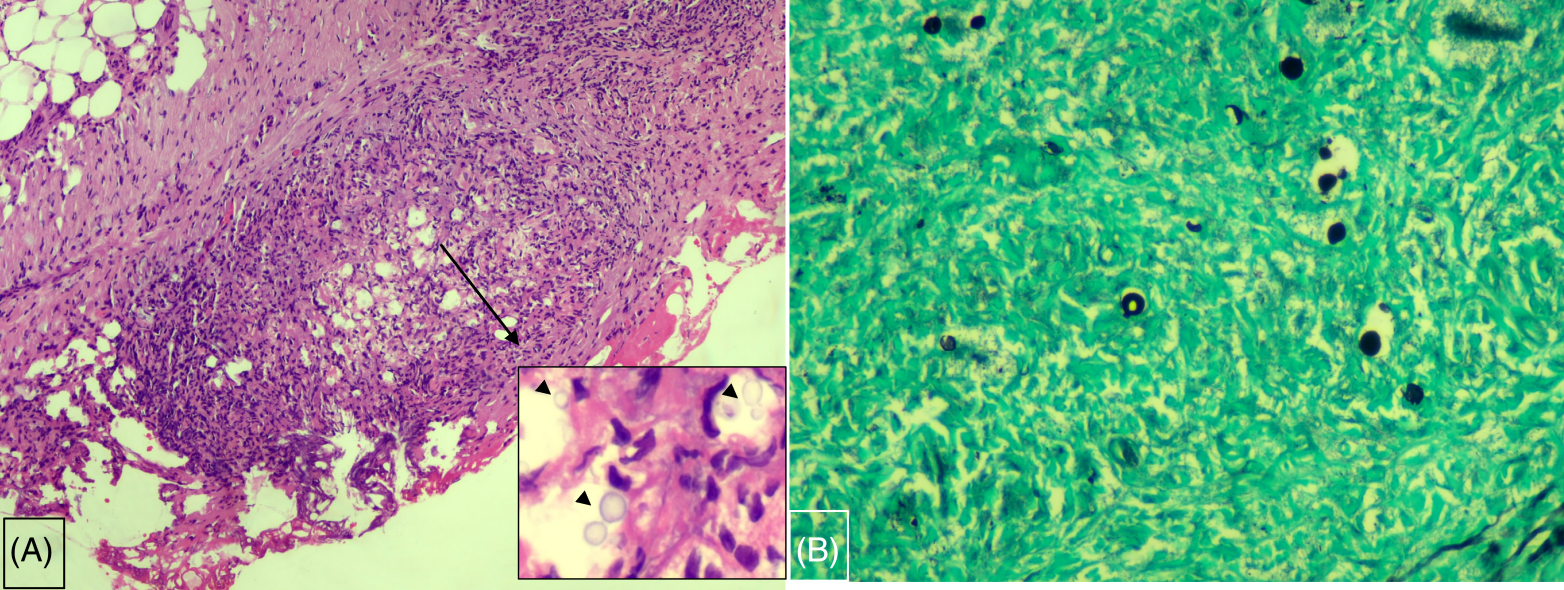
(A) Thoracoscopic biopsy specimen revealed granulomatous inflammation on H&E stain (⨉100). Inset window picture identified yeast organisms (arrow head) on H&E stain (⨉400). (B) Several black‐stained cryptococcus organisms were demonstrated on Grocott methenamine silver stain (⨉400).

For diagnostic confirmation, a surgical thoracoscopic pleural biopsy was performed, along with a repeated pleural fluid study. Thoracoscopy revealed multiple small nodules on the costal and diaphragmatic parietal pleura (Figure 1C). Simultaneous analysis of pleural fluid showed an exudate with WBC count 304/uL, MN predominance (97.1%), protein 4.1 g/dL, LDH 148 U/L, and ADA 36.7 IU/L (Table 1). The pleural fluid was positive for cryptococcal antigen, at a semi‐quantitative titre of 1:10. The pleural biopsy revealed chronic granulomatous inflammation on H&E stain (Figure 2A), and the Grocott methenamine silver stain on the biopsy specimen showed a positive result for *C. neoformans* (Figure 2B). The pleural fluid and tissue cultures were negative for *C. neoformans* and *M. tuberculosis*. His cerebrospinal fluid was negative for cryptococcal antigen, while the repeated serum cryptococcal antigen test was positive (Table 1).

The patient was treated with fluconazole 400 mg per day. Subsequently, his condition improved, and there was no reaccumulation of pleural effusion during the 1‐month follow‐up period while he remained on continuous medication.

## DISCUSSION

The current case presents isolated unilateral pleural effusion without an apparent pulmonary parenchymal lesion in a heart transplantation recipient receiving immunosuppressive drugs. MN‐predominant exudate, moderately elevated pleural fluid ADA levels, and focal pleural nodularity on chest CT were found. Finally, isolated cryptococcal pleural effusion was diagnosed based on a positive pleural fluid culture and antigen for *C. neoformans*, along with histological confirmation.

There are six cases in English‐language literature that measured pleural fluid ADA levels in cryptococcal pleural effusion (Table 1),^2^, ^3^, ^4^, ^5^, ^6^, ^7^ although more cases of cryptococcal pleural effusion have been reported.^1^, ^8^, ^9^, ^10^, ^11^ Males were absolutely predominant, and all of them showed exudate. Six cases with an available differential leukocyte count of pleural fluid, including our case, all showed MN predominance. Elevated pleural fluid ADA levels >40 IU/L were found in 3 (43%) out of seven cases.^5^, ^6^, ^7^ The sample size was inadequate for analysis; however, it appears that high pleural fluid ADA levels >40 IU/L did not correlate with any parameters, including age, underlying immunosuppressive condition, clinical presentation, and culture results. Because pleural fluid ADA levels have been used as an adjunctive diagnostic tool for TPE, empirical anti‐TB therapy based on this biomarker is often employed in clinical practice.^12^, ^13^ Two out of three cryptococcal pleuritis with ADA levels >40 IU/L were initially misdiagnosed as TPE.^5^, ^6^ Thus, physicians should be careful in making a presumptive diagnosis of TPE based on pleural fluid ADA levels, especially in immunocompromised hosts, which appear to be the most important clue to potential cryptococcal pleural effusion (Table 1).

In cases with a suspicion of cryptococcal pleural effusion, the cryptococcal antigen test of pleural fluid appears to be a valuable diagnostic tool for early diagnosis and treatment. Table 1 shows that the pleural fluid cryptococcal antigen test (100%) was more sensitive than both the pleural fluid culture (86%) and the serum cryptococcal antigen test (71%). In another previous case review, including cases without presented ADA values, it was also shown that the pleural fluid cryptococcal antigen test was positive in 8 (80%) out of 10 cases in which this test was performed.^10^ In contrast, the positivity rates for pleural fluid culture and serum antigen test were 42%–74% and 63%, respectively.^8^, ^9^, ^10^ Furthermore, the pleural fluid cryptococcal antigen test exhibited a high specificity.^8^, ^14^ Serum cryptococcal antigen can serve as an adjunctive screening test, although its sensitivity is not as high as that of pleural fluid cryptococcal antigen.^14^ However, even though our case showed positive results for pleural fluid cryptococcal antigen and culture, as well as a serum cryptococcal antigen test, these positive results were not consistently reproducible. The test results appear to be influenced by either the progression of the infection or its severity. Therefore, if there is a strong suspicion of this infection, it is necessary to repeat the cryptococcal antigen and culture tests.

Pleural nodularity on chest CT scan is a well‐known feature indicative of malignant pleural effusion.^15^ However, TPE can also manifest this focal nodular pleural thickening.^16^ The current cryptococcal case showed focal nodular pleural thickening compatible with that of TPE. To our knowledge, this CT finding represents the first documented occurrence of cryptococcal pleural effusion. Thus, our result suggests that any granulomatous pleural disease may present with these focal pleural thickening.

Fluconazole is the preferred drug for treating mild to moderate pulmonary cryptococcosis in immunocompromised patients, when there are no diffuse pulmonary infiltrates, disseminated infection, or meningeal involvement.^17^ Treatment consists of fluconazole at a dosage 400 mg once daily for 6–12 months. Acceptable alternatives include itraconazole, voriconazole, or posaconazole. In the context of this recommendation, our case, presenting with isolated cryptococcal pleural infection, was treated with daily oral fluconazole and showed clinical and radiological improvement.

In conclusion, cryptococcal infection should be considered as a potential aetiology in immunosuppressed patients with an exudative pleural effusion of unknown cause, even in the presence of elevated ADA levels or focal pleural thickening on CT. In addition to the routinely performed pleural fluid culture, the pleural fluid cryptococcal antigen test, as well as serum cryptococcal antigen test, play useful diagnostic roles in this context. Repeated thoracentesis and pleural biopsy may be required for definite confirmation of this unusual aetiology.

## AUTHOR CONTRIBUTIONS

**JL**: Conceptualization; writing‐review. **BEP**: Methodology; writing‐review. **SYJ**: Methodology; writing‐review. **CHK**: Conceptualization; writing‐original draft; supervision. All authors critically reviewed the manuscript and approved the final version.

## CONFLICT OF INTEREST STATEMENT

None declared.

## ETHICS STATEMENT

The authors declare that appropriate written informed consent was obtained for the publication of this manuscript and accompanying images.

## Data Availability

The data that support the findings of this study are available on request from the corresponding author. The data are not publicly available due to privacy or ethical restrictions.
